# The Complete Chloroplast Genome of *Meconopsis simplicifolia* and Its Genetic Comparison to Other *Meconopsis* Species

**DOI:** 10.3390/genes15101301

**Published:** 2024-10-06

**Authors:** Min Sun, Zhidan Zhu, Rui Li

**Affiliations:** 1 Institute of Advanced Study, Chengdu University, Chengdu 610106, China; summin1028@163.com; 2 Engineering Research Center of Sichuan-Tibet Traditional Medicinal Plant, Chengdu 610000, China; 19981499702@169.com; 3 School of Food and Biological Engineering, Chengdu University, Chengdu 610106, China

**Keywords:** chloroplast genome, *Meconopsis*, *Meconopsis simplicifolia*, phylogenetic analysis, comparative genomics

## Abstract

**Background:** Chloroplasts, due to their high conservation and lack of recombination, serve as important genetic resources for the classification and evolutionary analysis of closely related species that are difficult to distinguish based on their morphological features. *Meconopsis simplicifolia (M. simplicifolia)*, an endangered herb within the *Meconopsis* genus, has demonstrated therapeutic potential in treating various diseases. However, the highly polymorphic morphology of this species poses a challenge for accurate identification. **Methods:** In this study, the complete chloroplast genome of *M. simplicifolia* was sequenced and assembled using Illumina sequencing technology. Simple sequence repeats (SSRs) and repetitive sequences were characterized. In addition, a comparative analysis was conducted with the chloroplast genomes of six other *Meconopsis* species. **Results:** The chloroplast genome of *M. simplicifolia* has a quadripartite circular structure with a total length of 152,772 bp. It consists of a large single-copy region of 83,824 bp and a small single-copy region of 17,646 bp, separated by a pair of inverted repeat sequences (IRa and IRb, 25,651 bp). The genome contains 131 genes, 33 SSRs, and 27 long repetitive sequences. Comparative analysis with six other chloroplast genomes of *Meconopsis* revealed that *M. simplicifolia* is closely related to *M. betonicifolia* and that the *rpl2* (*ribosomal protein L2*) gene in the IRb region has been deleted. This deletion is of significant importance for future taxonomic studies of *M. simplicifolia*. **Conclusions:** This study provides a valuable reference for the identification of *M. simplicifolia* and contributes to a deeper understanding of the phylogeny and evolution of the *Meconopsis* genus.

## 1. Introduction

Chloroplasts are unique organelles in plants that play a crucial role in photosynthesis, growth and development, signal transduction under various stresses, and the biosynthesis of important metabolites [1,2]. Decoding the chloroplast genome is fundamental to understanding chloroplast function and its biological processes, which are of great significance for exploring the mechanisms of plant growth and stress response. Previous studies have shown that chloroplast deoxyribonucleic acid (DNA) typically has a quadripartite circular structure, a linear structure, or a multi-branch linear structure [3,4,5,6]. However, due to differences in plant species, cell developmental stages, and tissue types, it remains unclear which form of chloroplast DNA is more prevalent [7,8]. The size of chloroplast genomes ranges from 15,553 bp (*Asarum minus*) to 521,168 bp (*Floydiella terrestris*), and are typically divided into two regions: a large single-copy (LSC) region and a small single-copy (SSC) region [9,10], separated by two inverted repeats (IRa and IRb). The chloroplast genome generally contains between 101 and 118 genes, including approximately 80 protein-coding genes, four ribosomal ribonucleic acids (RNAs), and 30 transfer RNAs [11,12,13]. With advances in sequencing technology, obtaining a complete chloroplast genome has become more accessible and cost-effective, providing a valuable opportunity to explore the role of chloroplasts in plant biology.

Chloroplasts are maternally inherited organelles, and their structure is relatively conserved [10]. Compared with other plastids, such as nuclear and mitochondrial genomes, chloroplast genomes are characterized by their small size, simple structure, and moderate evolutionary rate [14,15]. Thus, chloroplasts not only play an important role in plant biology but also serve as valuable resources in the study of plant systematics and genetic relationships [16,17,18]. For example, chloroplast genome sequencing was performed on *Oreomecon nudicaulis*, a species with an unclear classification (originally assigned to the *Papaver* genus, it is now classified under *Oreomecon*). The analysis revealed that it is closely related to *Meconopsis* within the *Papaveraceae* family but does not form a clade with the *Papaver* genus, which is consistent with the revised classification [19]. Similarly, phylogenetic analysis of the chloroplast genome of the medicinal plant *Hypecoum erectum* L. showed its association with *H. zhukanum*, both of which belong to the *Hypecoideae* subfamily, a monophyletic group [20]. Moreover, structural variations in the chloroplast genome, such as gene deletions, large inversions, and the contraction or elongation of inverted repeat (IR) regions, provide important genetic information for the identification of specific plants [11]. Ren et al. found that, compared with other plants in the *Papaveroideae* subfamily, five typical genes located in the SSC region in the chloroplast genomes of *Corydalis saxicola* and *Corydalis tomentella* migrated to the IR region, resulting in IR elongation and gene duplication [21]. Additionally, polymorphisms were observed in the gap regions of seven genes, and coding polymorphisms were detected in three genes in the chloroplast genomes of *Papaveroideae* plants, indicating their potential to serve as molecular markers for phylogenetic and species identification studies [22]. In summary, chloroplast genome information provides an important basis for inferring evolutionary relationships in species and specific taxa.

*Meconopsis* is a genus of herbaceous plants, belonging to the *Papaveraceae* subfamily [23]. Globally, 49 known species of *Meconopsis* have been identified, several of which have demonstrated therapeutic efficacy [24]. These plants are mainly distributed across the Qinghai-Tibet Plateau, Hengduan Mountains, and Himalayan region at altitudes ranging between 2000 and 5800 m [25]. Geographic isolation and natural selection have promoted speciation, resulting in diverse *Meconopsis* species, making it challenging to classify them solely based on their phenotypic characteristics [26]. The complete chloroplast genome provides a powerful tool for the accurate identification and classification of species [27]. In this study, the chloroplast genome of the endangered plant *M. simplicifolia* was sequenced using second-generation sequencing technology and a complete chloroplast genome information of 152,772 bp containing 131 genes was obtained. A comparative analysis was conducted to determine the phylogenetic relationship between *M. simplicifolia* and other *Meconopsis* species; it was confirmed that *M. simplicifolia* and *M. betonicifolia* have a closer phylogenetic relationship. The regions LSC and SSC are highly variable in the chloroplast genomes of *Meconopsis* species. This study is of profound significance for the identification of *M. simplicifolia*, the exploration of its chloroplast function, and improvement in the phylogenetic understanding within the *Meconopsis* genus.

## 2. Materials and Methods

### 2.1. Plant Material

**Figure 1:**
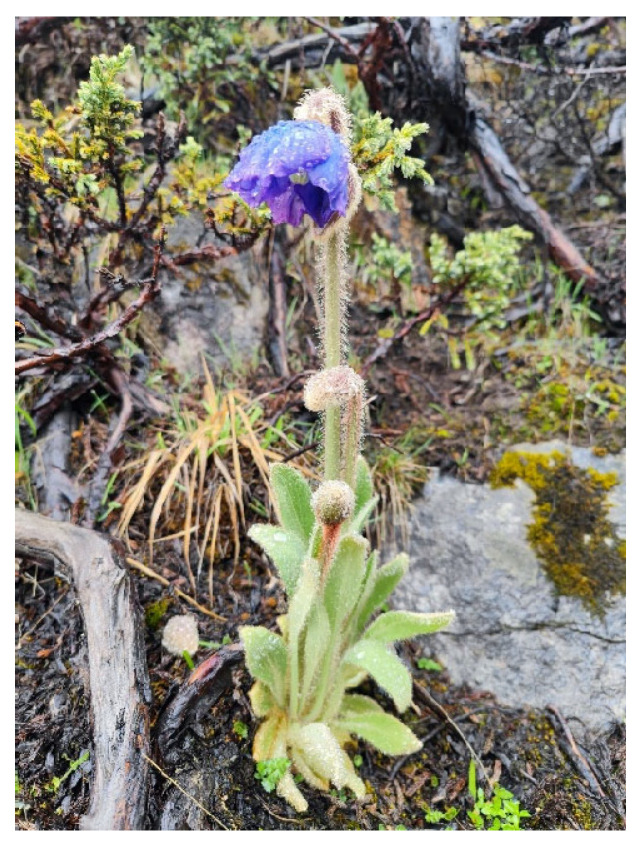
A photograph of *M. simplicifolia* in the flowering stage.

Considering the distribution of *M. simplicifolia*, one individual was collected from the wild at Lingzhi, Tibet, China (38.9784° N, 105.9035° E) in July 2022. A voucher specimen (voucher number: NMU00912) was deposited at the Herbarium of North Minzu University (Figure 1). The collection of plant material adhered to the relevant institutional, national, and international guidelines and legislation, and we obtained permission to collect the *M. simplicifolia*.

### 2.2. Sequencing, Assembly, and Annotation of the M. simplicifolia cp Genome

Genomic DNA was extracted from the fresh leaves using the DNeasy Plant Mini Kit (Qiagen, Redwood City, CA, USA), following the manufacturer’s protocol. A paired-end library with an insertion size of 350 bp was constructed according to the manufacturer’s instructions and sequenced using the Illumina HiSeq 2500 platform. Approximately 5.57 Gb of raw reads were generated and assembled into non-redundant contigs using NOVOPlasty [28], a de novo sequence assembly software package, with k = 39 and a genome range of 120,000–200,000. Initial gene annotation was performed using Plann [29] with the chloroplast genome of *M. racemosa* (GenBank accession number: MK533649) as the reference genome [30], and the annotation was refined using Geneious software (https://www.geneious.com/, accessed on 1 January 2024) [31].

### 2.3. SSRs and Repeated Sequences

The Perl script MISA v2.1 (http://pgrc.ipk-gatersleben.de/misa/misa.html, accessed on 3 January 2024) was applied to detect simple sequence repeats (SSRs) in the cp genome with the settings as follows: 10 for mono-, 5 for di-, 4 for tri-, and 3 for tetra-, penta- and hexanucleotide. Repeats (forward, palindrome, complement, and reverse sequences) were identified using online REPuter software (https://bibiserv.cebitec.uni-bielefeld.de/reputer?id=reputer_view_submission, accessed on 3 January 2024), with the default settings.

### 2.4. Comparative Analysis of cp Genome Structure

Whole-genome comparative analysis was conducted on the cp genomes of the following seven *Meconopsis* species: *M. simplicifolia* (this study, NC_070211), *M. horridula* (MK533646) [30], *M. integrifolia* (MK533647) [30], *M. punicea* (MK533648) [30], *M. racemosa* (MK533649) [30], *M. henrici* (MN488591) [32], and *M. quintuplinervia* (MK801686) [33]. Comparative genomic analysis of *M. simplicifolia* and six other *Meconopsis* species was performed using BLAST Ring Image Generator (BRIG) software [34]. Meanwhile, the comparison and variation in the cp genomes’s architecture were visualized using the Shuffle-LAGAN mode of the mVISTA software (https://genome.lbl.gov/vista/index.shtml, accessed on 3 January 2024) [35]. The IRscope tool (https://irscope.shinyapps.io/irapp/, accessed on 3 January 2024) was used to compare and illustrate the IR border regions in the seven *Meconopsis* species [36].

### 2.5. Phylogenetic Analysis

Phylogenetic relationships were reconstructed based on the 10 cp genomes, using *Papaver orientale* (NC_037832) [37] from the NCBI database as the ‘outgroup’. The coding sequences of the protein-coding genes present in all the cp genomes of the *Papaveraceae* species were aligned using MAFFT-LINSI v7.313 [38]. The optimal trees were inferred by maximum likelihood phylogenetic analysis using RAxML v8.2.11 [39] with the GTRGAMMA model and 500 bootstrap replicates.

## 3. Results

### 3.1. Features of the cp Genome of Meconopsis simplicifolia

**Figure 2:**
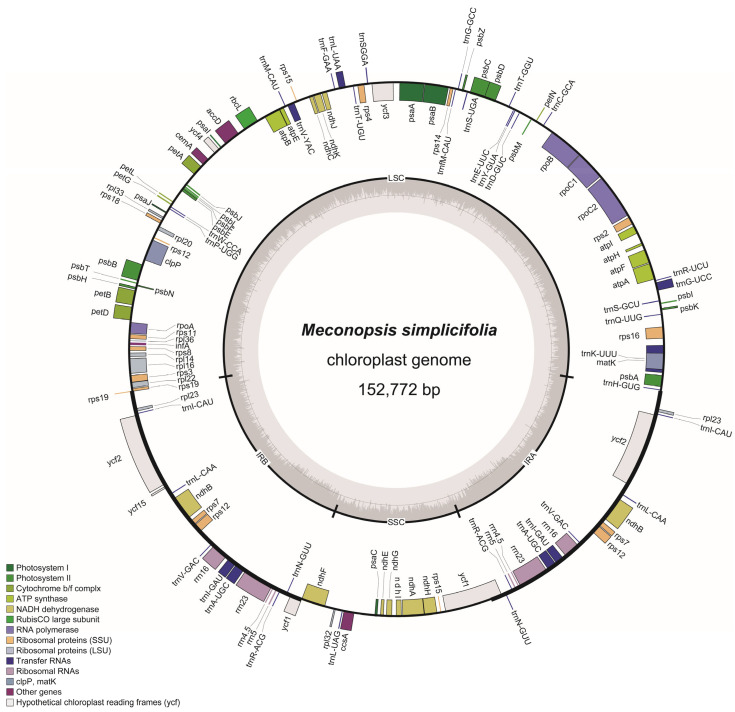
A map of the chloroplast genome of *M. simplicifolia*. The genes inside and outside the circle are transcribed in a clockwise and counterclockwise direction, respectively. Genes belonging to different functional groups are shown in different colors. The darker and lighter grey in the inner circle each represent GC and AT content.

The assembled cp genome of *M. simplicifolia* has been deposited in GenBank with the accession number NC_070211. The cp DNA of *M. simplicifolia* measured 152,772 bp in length and exhibited the typical quadripartite structure (Table 1 and Figure 2). It consisted of a pair of inverted repeat regions (IRa and IRb) spanning 25,651 bp each, along with separate single-copy regions, including the SSC of 17,646 bp and the LSC of 83,824 bp. The *M. simplicifolia* cp genome contained 131 predicted functional genes, comprising 84 protein-coding genes, 37 tRNA genes, 8 rRNA genes, and 2 pseudogenes (*rps15* and *rps19*) (Table 2). There were 21 intron-containing genes, of which 13 were protein-coding genes and 8 were tRNA genes. Out of the intron-containing genes, 18 contained a single intron, while 3 (*clpP*, *rps12*, and *ycf3*) contained two introns.

The overall GC content of the *M. simplicifolia* cp genome was 38.74%. Regarding the protein-coding regions, the GC content of the first, second, and third codons was 45.91%, 38.17%, and 31.15%, respectively. The 84 protein-coding genes encoded 25,454 codons, with leucine (L) the most frequently used amino acid (10.36%), and cysteine (C) the least frequently used (1.19%).

### 3.2. SSRs and Long-Repeat Analysis

**Figure 3:**
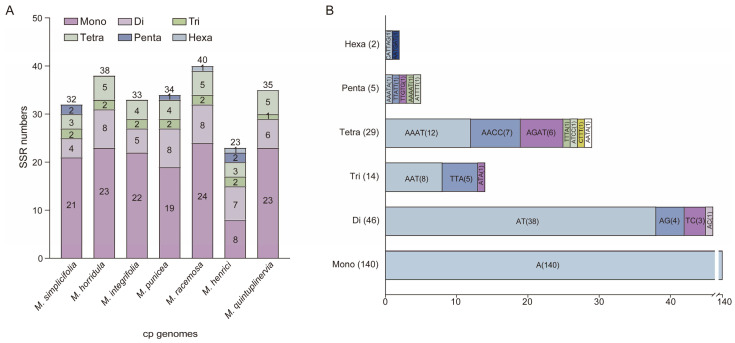
An analysis of simple sequence repeats (SSRs) in the cp genomes of the seven *Meconopsis* species. (**A**) Number of different types of SSRs; (**B**) frequency of identified SSR motifs in different repeat classes.

SSRs, also known as microsatellites, are tandem repeat sequences consisting of 1–6 nucleotide repeat units. They are widely distributed in cp genomes and often used as genetic markers in population genetics and evolutionary studies due to their high intraspecific variability [40]. In this study, we analyzed SSRs in the cp genomes of seven *Meconopsis* species. A total of 33 SSRs were identified in the *M. simplicifolia* cp genome. Similarly, *M. horridula*, *M. integrifolia*, *M. punicea*, *M. racemose*, *M. henrici*, and *M. quintuplinervia* contained 38, 33, 34, 40, 23, and 35 SSRs, respectively (Figure 3A, Table S1). Among all chloroplast genomes, mononucleotide repeats were the most frequent, ranging between 8 and 24, accounting for 34.78% (8/23) to 66.67% (24/33) of all SSRs, followed by dinucleotide, ranging between 4 (12.12%, 4/33) and 8 (23.35%, 8/34), tetranucleotide, ranging between 3 (9.09%, 3/33) and 5 (14.29%, 5/35), trinucleotide, ranging between 1 (2.86%, 1/35) and 3 (9.09%, 3/33), pentanucleotide, ranging between 0 and 2 (8.70%, 2/23), and pentanucleotide, ranging between 0 and 1 (4.35%, 1/23). In *M. simplicifolia*, all mononucleotide repeats (100%) and the majority of dinucleotide repeats (75%) consisted of A/T nucleotides (Figure 3B).

**Figure 4:**
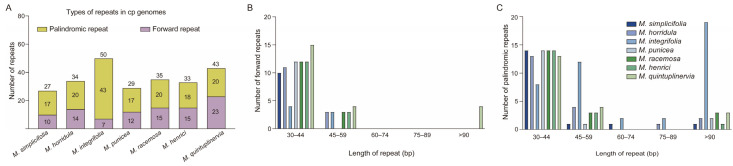
An analysis of the repeated sequences in the cp genomes of the seven *Meconopsis* species. (**A**) Frequency of palindromic and forward repeats; (**B**) frequency of forward repeats by length; (**C**) frequency of palindromic repeats by length.

Repeat sequences play a crucial role in phylogenetic research and genome reorganization. In the cp genome of *M. simplicifolia*, 27 dispersed repeats were identified, including 10 forward repeats and 17 palindromic repeats (Figure 4A). This pattern is consistent with the other six *Meconopsis* cp genomes, with the number of repeats ranging between 29 in *M. punicea* and 50 in *M. integrifolia*. Palindromic repeats were the most prevalent repeat type among the seven *Meconopsis* species (Figure 4B,C). Most of these repeats ranged between 30 bp and 44 bp in length.

### 3.3. Comparative Analysis of cp Genomes of Meconopsis Species

**Figure 5:**
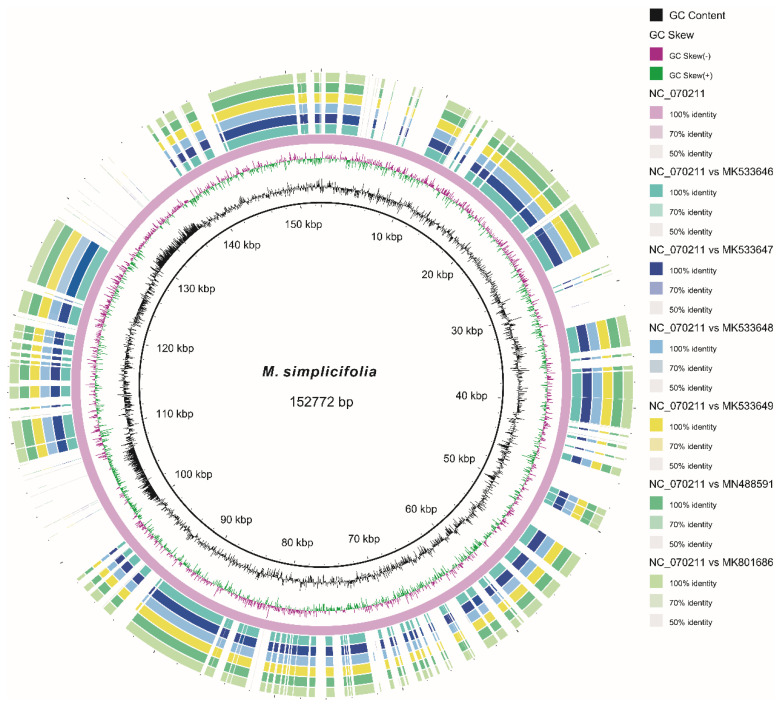
BRIG ring comparison of the cp genomes of the seven *Meconopsis* species. The innermost rings represent the GC content (black) and GC skew (purple/green). The seven outer circles depict the similarity results compared to the reference genome (*M. simplicifolia*. NC_070211).

**Figure 6:**
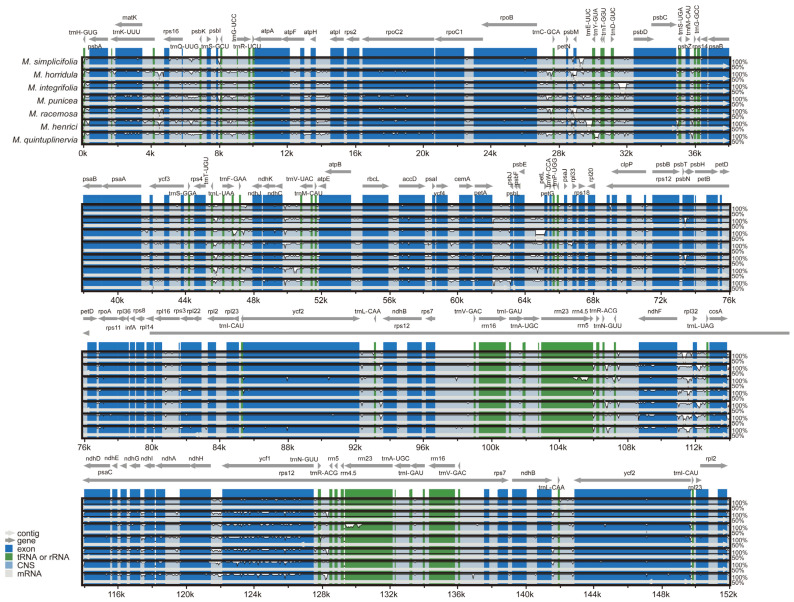
Alignment of the cp genomes of the seven *Meconopsis* species. The white regions show the sequence differences among all analyzed chloroplast genomes. The horizontal axis shows the positions within the chloroplast genome, and the vertical scale indicates the identity percentage, ranging between 50% and 100%.

A comparative analysis of cp genomes provided valuable insights into intricate evolutionary relationships. In this study, we compared the cp genomes of *M. simplicifolia* and other six *Meconopsis* species. The size of the seven *Meconopsis* cp genomes ranged between 151,864 (*M. integrifolia*) and 154,997 bp (*M. quintuplinervia*). Notably, within the genus *Meconopsis*, the genome of *M. simplicifolia* exhibited a higher degree of conservation and could be accurately mapped (Figure 5). The sequence consistency of *Meconopsis* cp genomes was further evaluated using mVISTA software. The results revealed that the IR regions exhibited fewer differences compared to the LSC and SSC regions (Figure 6). Non-coding regions displayed more variability than coding regions, with significant changes observed in the intergenic spacers among the seven cp genomes. These highly divergent regions included *trnH-psbA*, *matK*, *rps16-psbK*, *atpH-atpI*, *rpoC2*, *psbM-petN*, *trnE-trnT*, *trnT-psbD*, *psaA-ycf3*, *trnF-ndhJ*, *ndhK*, *ndhC-trnV*, *atpB-rbcL*, *accD*, *ycf4-cemA*, *petA-psbL*, *psbE-petL*, *clpP-psbB*, *rpl16*, *ndhF-rpl32-ccsA*, and *ycf1*, among others.

**Figure 7:**
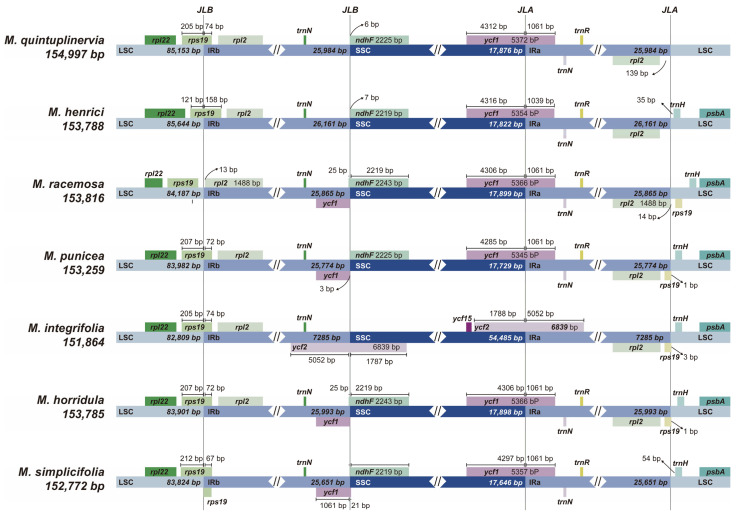
A comparison of the border regions of LSC, SSC, and IR regions among the cp genomes of the seven *Meconopsis* species.

A detailed comparison of the binding regions between the inverted repeats (IR/LSC and IR/SSC) was performed among the seven *Meconopsis* species (Figure 7). In all species, the *rpl22* gene was located within the LSC region. Variations in gene content and order were observed, such as the presence of the *ycf1* gene at the SSC/IRa junction in *M. simplicifolia, M. horridula, M. punicea, M. racemose, M. henrici*, and *M. quintuplinervia*, while *M. integrifolia* had a missing *ycf1* gene in the SSC/IRa junction. Expansion and contraction of the inverted repeat region were observed. For example, the *rps19* gene was found within the LSC region in *M. racemose*, while in the other six *Meconopsis* species, it was located 67–158 bp away, spanning the LSC and IRb binding regions. Except for *M. simplicifolia*, the *rpl2* gene did not extend into the LSC region in the other species. Overall, there were only minor variations in the IR boundary regions among the cp genomes of these seven *Meconopsis* species.

### 3.4. Phylogenetic Analysis of M. simplicifolia and Related Meconopsis species cp Genomes

**Figure 8:**
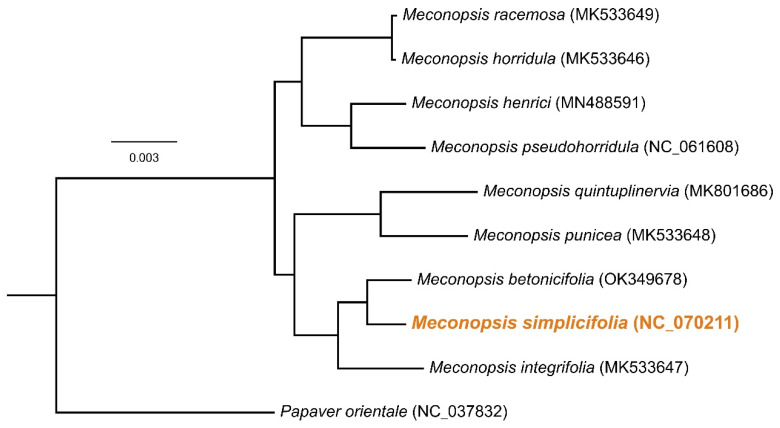
**A** species tree based on the coding sequences of 75 protein-coding genes shared by the cp genomes of 10 Papaveraceae members including *M. simplicifolia* (NC_070211; this study), *M. horridula* (MK533646) [30], *M. integrifolia* (MK533647) [30], *M. punicea* (MK533648) [30], *M. racemosa* (MK533649) [30], *M. henrici* (MN488591) [32], *M. quintuplinervia* (MK801686) [33], *M. pseudohorridula* (NC_061608; unpublished), *M. betonicifolia* (OK349678; unpublished), and the outgroup species *Papaver orientale* (NC_037832) [37]. All nodes received 100% bootstrap support. Orange represents *M Simplicifolia* chloroplast genome.

To determine the phylogenetic position of *M. simplicifolia* within the Papaveraceae family, we utilized the cp genomes of ten *Papaveraceae* members, including *M. simplicifolia*. A species tree was constructed based on the alignment of 75 shared protein-coding genes from the cp genomes. The analysis revealed that *M. simplicifolia* clustered together with *M. betonicifolia* (Figure 8). All *Meconopsis* plants formed a well-supported branch, indicating the high potential of cp genomes for species differentiation within the order Papaveraceae.

## 4. Discussion

The cp genome serves as a valuable resource for interspecific differentiation and various biotechnological applications [41]. In this study, we sequenced and assembled the complete cp genome of *M. simplicifolia*. The cp genome of *M. simplicifolia* has a typical quadripartite circular structure with a GC content of 38.74%, similar to the chloroplast genome characteristics of most *Meconopsis* species [1]. The size of the *M. simplicifolia* cp genome was 152,772 bp, consistent with other *Meconopsis* species ranging between 151 and 154 kb [30,32,33]. All these complete cp genomes displayed a GC content of 38%, which is in line with the low GC content observed in the cp genomes of other angiosperms [13].

SSRs have been extensively used in phylogenetic relationships, genetic diversity studies, and species identification. The SSRs identified in the cp genomes of seven *Meconopsis* were mostly composed of mononucleotide repeats consisting mainly of T, which is consistent with previous studies on *Meconopsis* species [30]. The availability of genomic resources in *Meconopsis* plants can enhance the understanding of population patterns and the identification of gene regions associated with important medicinal and environmental adaptive traits [30]. SSRs provide effective marker resources for species identification and genetic diversity studies of *Meconopsis* and related species. Repeat sequences have the potential to promote chloroplast genome rearrangement and increase population genetic diversity, and are widely used to identify mutation hotspots and establish phylogenetic relationships [42]. In this study, we identified 27 repeat sequences in *M. simplicifolia*, which is fewer compared to other *Meconopsis* species. Most of these repeats were located in genes, indicating that the cp genome of *M. simplicifolia* retains a significant amount of genetic material.

The analysis of protein-coding genes revealed that *M. simplicifolia* shares 75 genes with the other six *Meconopsis* species. Similar to other *Meconopsis* species, *M. simplicifolia* lacks the *rpl2* gene, which plays a crucial role in chloroplast development during early leaf development [43]. It is hypothesized that the *Msrpl2* gene has either been functionally transferred to the nucleus or replaced by a eukaryotic gene. Comparative analysis of cp genomes using BRIG and mVISTA showed a high sequence identity among all *Meconopsis* species. However, *Meconopsis* cp genomes have also undergone gene duplication, gene/intron loss, insertion/deletion, pseudogenization, and varying expansion/contraction of the inverted repeat region. These genomic events are consistent with observations in other angiosperms [12,13]. In *M. simplicifolia*, the pseudogenization of *rps19* in the IR regions is consistent with a previous study [30]. The sequence and content of the SC regions show less similarity compared to the IR regions in *Meconopsis* species. The most highly divergent non-coding regions were identified in the intergenic regions of *trnT-psbD*, *ndhC-trnV*, and *ndhF-rpl32-ccsA*, which have also been recognized as molecular markers in many land plants [44,45].

Taxonomy and phylogeny of *Meconopsis* have been extensively studied at the genus level [46,47]. Previous studies on the evolutionary relationships among different *Meconopsis* species utilized internal transcribed spacer and cpDNA sequences, as well as amplified fragment length polymorphisms (AFLPs) [46,47,48]. However, complete genome sequencing offers a more comprehensive perspective [49]. In the case of *M. simplicifolia*, limited information was available. The phylogenetic position of *M. simplicifolia* within *Meconopsis* was determined using cp genomes and 75 protein-coding genes among nine *Meconopsis* species. Phylogenetic analysis revealed that all *Meconopsis* species formed a monophyletic clade with 100% bootstrap support. *M. simplicifolia* was found to be closely related to *M. betonicifolia*, supporting previous morphological and molecular data [46].

## 5. Conclusions

This study characterized the complete cp genome of *M. simplicifolia* and conducted a comparative analysis of other six *Meconopsis* cp genomes, revealing the conserved genome structure and organization across seven *Meconopsis* species. The most divergent regions among these *Meconopsis* cp genomes were identified in three non-coding intergenic spacer (IGS) regions (*trnT-psbD*, *ndhC-trnV*, and *ndhF-rpl32-ccsA*) and three genic regions (*matK*, *rpoC2*, and *ycf1*). Moreover, the genetic resources such as SSRs and repetitive sequences discovered in the cp genomes can serve as valuable molecular markers for the identification of *Meconopsis* species. Phylogenetic analysis demonstrated that *M. simplicifolia* is closely related to *M. betonicifolia*. The comprehensive cp genome provides essential resources for genetic and biological studies of *Meconopsis* and other species within the *Papaveraceae* family.

## Figures and Tables

**Table 1:** A summary of the complete chloroplast genomes of seven *Meconopsis* species.

Feature	*M. simplicifolia*	*M. horridula*	*M. integrifolia*	*M. punicea*	*M. racemosa*	*M. henrici*	*M. quintuplinervia*
Accession number	NC_070211	MK533646	MK533647	MK533648	MK533649	MN488591	MK801686
Genome size (bp)	152,772	153,785	151,864	153,259	153,816	153,388	154,997
IR length (bp)	25,651	51,988	51,306	51,548	51,988	26,107	25,984
SSC length (bp)	17,646	17,898	17,749	17,729	17,898	17,822	17,876
LSC length (bp)	83,824	83,899	82,809	83,982	83,930	83,698	85,153
No. of total genes	131	127	127	127	127	112	129
No. of protein-coding genes	84	90	90	90	90	78	84
No. of tRNA genes	39	37	37	37	37	30	37
No. of rRNA genes	8	8	8	8	8	3	8
Overall GC content (%)	38.7	38.8	38.8	38.5	38.7	38.5	38.5

**Table 2:** A list of the genes in the *M. simplicifolia* chloroplast genome.

Category	Gene Group	Gene Name
Self-replication	Ribosomal protein (large subunit) (9)	*rpl14, rpl16* ^a^, *rpl20*, *rpl22*, *rpl23* ^b^, *rpl32*, *rpl33*, *rpl36*
	Ribosomal protein (small subunit) (16)	*rps2*, *rps3*, *rps4*, *rps7* ^b^, *rps8*, *rps11*, *rps12* ^a,b^, *rps14*, *rps15*, *rps16* ^a^, *rps18*, *rps19*
	DNA-dependent RNA polymerase (4)	*rpoA*, *rpoB*, *rpoC1* ^a^, *rpoC2*
	rRNA genes (8)	*rrn16* ^b^, *rrn23* ^b^, *rrn4.5* ^b^, *rrn5* ^b^,
	tRNA genes (37)	*trnH-GUG*, *trnK-UUU* ^a^, *trnQ-UUG*, *trnS-GCU*, *trnG-UCC* ^a^, *trnR-UCU*, *trnC-GCA*, *trnD-GUC*, *trnY-GUA*, *trnE-UUC*, *trnT-GGU*, *trnS-UGA*, *trnG-GCC*, *trnS-GGA*, *trnT-UGU*, *trnL-UAA* ^a^, *trnF-GAA*, *trnV-UAC* ^a^, *trnfM-CAU* ^b^, *trnW-CCA*, *trnP-UGG*, *trnI-CAU* ^b^, *trnL-CAA* ^b^, *trnV-GAC* ^b^, *trnI-GAU* ^a,b^, *trnA-UGC* ^a,b^, *trnR-ACG* ^b^, *trnN-GUU* ^b^, *trnL-UAG*
Photosynthesis	Photosystem I (5)	*psaA*, *psaB*, *psaC*, *psaI*, *psaJ*
	Photosystem II (15)	*psbA*, *psbB*, *psbC*, *psbD*, *psbE*, *psbF*, *psbH*, *psbI*, *psbJ*, *psbK*, *psbL*, *psbM*, *psbN*, *psbT*, *psbZ*
	NADH dehydrogenase (11)	*ndhA* ^a^, *ndhB* ^a,b^, *ndhC*, *ndhE*, *ndhF*, *ndhG*, *ndhH*, *ndhI*, *ndhJ*, *ndhK*
	Cytochrome b/f complex (6)	*petA*, *petB* ^a^, *petD* ^a^, *petG*, *petL*, *petN*
	ATP synthase (6)	*atpA*, *atpB*, *atpE*, *atpF* ^a^, *atpH*, *atpI*
	Large subunit of rubisco (1)	*rbcL*
Other genes	Translational initiation factor (1)	*infA*
	ATP-dependent protease subunit p gene (1)	*clpP* ^a^
	Maturase (1)	*matK*
	Envelope membrane protein (1)	*cemA*
Unknown	Subunit of acetyl-CoA-carboxylase (1)	*accD*
	C-type cytochrome synthesis gene (1)	*ccsA*
	Conserved hypothetical chloroplast ORF (7)	*ycf1* ^b^, *ycf2* ^b^, *ycf3* ^a^, *ycf4*, *ycf15*,
	Pseudogene (2)	*rps15*, *rps19*

Notes: ^a^ Genes containing introns; ^b^ two gene copies in IR.

## Data Availability

The genome sequence data that support the findings of this study are openly available in GenBank of NCBI at https://www.ncbi.nlm.nih.gov/ (accessed on 15 February 2023), under the accession no. NC_070211. The associated BioProject, SRA, and Bio-Sample numbers are PRJNA935484, SRR23491443, and SAMN33317127, respectively.

## Supplementary Materials

The following supporting information can be downloaded at: https://www.mdpi.com/article/10.3390/genes15101301/s1, Table S1. Types and numbers of SSRs in the chloroplast genomes of *M. simplicifolia*, *M. horridula*, *M. integrifolia*, *M. punicea*, *M. racemose*, *M. henrici*, and *M. quintuplinervia*.
