# Supplementary material for: The Complete Chloroplast Genome of Meconopsis simplicifolia and Its Genetic Comparison to Other Meconopsis Species
Source: Genes (Basel). 2024 Oct 6;15(10):1301. doi: 10.3390/genes15101301 (PMC11507337; doi:10.3390/genes15101301)
Supplement: Supplementary file 1 [file genes-15-01301-s001.zip › genes-3230636-supplementary.pdf]

Table S1

Types and numbers of SSRs in the chloroplast genomes of *M. simplicifolia*, *M. horridula, M. integrifolia*, *M. punicea*, *M. racemose*, *M. henrici*, and *M. quintuplinervia*

SSR type	Repeat unit	*M. simplicifolia*	*M. horridula*	*M. integrifolia*	*M. punicea*	*M. racemosa*	*M. henrici*	*M. quintuplinervia*	Total
Mono	A/T	21	23	22	19	24	8	23	140
Di	AG/CT	0	1	1	1	1	0	0	4
	AC/GT	0	0	0	1	0	0	0	1
	TC/GA	1	0	0	0	0	1	1	3
	AT/AT	3	7	4	6	7	6	5	38
Tri	AAT/ATT	0	2	2	2	2	0	0	8
	TTA/TAA	2	0	0	0	0	2	1	5
	ATA/TAT	1	0	0	0	0	0	0	1
Tetra	AAAT/ATTT	1	3	2	2	3	0	1	12
	AACC/GGTT	1	1	1	1	1	1	1	7
	AGAT/ATCT	1	1	1	0	1	1	1	6
	TTTA/TAAA	0	0	0	0	0	1	0	1
	ATCC/ATGG	0	0	0	1	0	0	0	1
	CTTT/AAAG	0	0	0	0	0	0	1	1
	AATA/TATT	0	0	0	0	0	0	1	1
Penta	AAATA/TATTT	1	0	0	0	0	0	0	1
	TTATT/AATAA	1	0	0	0	0	0	0	1
	TTGTG/CACAA	0	0	0	0	0	1	0	1
	AAAAT/ATTTT	0	0	0	1	0	0	0	1
	ATTTT/AAAAT	0	0	0	0	0	1	0	1
Hexa	CATTAG/CTAATG	0	0	0	0	0	1	0	1
	AATGAT/ATCATT	0	0	0	0	1	0	0	1
	Total	33	38	33	34	40	23	35	236
